# Giant mesenteric cyst of mesothelial origin as a rare late complication of previous peritoneal dialysis during hemodialysis 

**DOI:** 10.5414/CNCS112023

**Published:** 2026-05-29

**Authors:** Sarra Hadded, Chayma Soltani, Wael Ferjaoui, Yosra Ben Ariba, Jannet Laabidi

**Affiliations:** 1 Department of Nephrology, Mongi Slim Hospital,; 2 Department of Nephrology,; 3 Department of General Surgery, Military Hospital of Tunis, and; 4 Faculty of Medicine, University of Tunis El Manar, Tunis, Tunisia

**Keywords:** mesenteric cyst, hemodialysis, peritoneal dialysis, peritonitis

## Abstract

Mesenteric cysts are uncommon benign abdominal lesions with no specific clinical features. Their occurrence during hemodialysis is exceptional. We report the case of a 22-year-old patient who had been treated with peritoneal dialysis for 2 years and was subsequently transferred to hemodialysis because of refractory peritonitis. Three years later, he presented with a progressive abdominal distension caused by a large mass extending from the epigastric to the pelvic region. Exploratory laparoscopy revealed a giant cystic lesion occupying the entire peritoneal cavity. Complete surgical excision was performed. Histopathological examination confirmed a benign mesenteric cyst of mesothelial origin. This rare complication may be associated with previous peritoneal dialysis, recurrent peritonitis, and a chronic inflammatory state.

## Introduction 

Mesenteric cysts are rare benign peritoneal tumors characterized by the formation of intra-abdominal cystic masses arising from the mesentery [1]. Their etiopathogenesis remains poorly understood. In patients undergoing peritoneal dialysis, peritoneal pseudocysts may develop, particularly following episodes of peritonitis [2]. However, the occurrence of a mesenteric cyst of mesothelial origin during dialysis is exceptional. Here, we report a case of a giant mesenteric cyst diagnosed in a hemodialysis patient, which may be associated with a persistent inflammatory state and a history of peritoneal dialysis complicated by refractory peritonitis. 

## Case presentation 

We report the case of a 22-year-old man with end-stage renal disease secondary to hereditary chronic glomerular nephropathy. He had been on peritoneal dialysis for 2 years before being transferred to hemodialysis due to refractory episodes of peritonitis caused by methicillin-resistant Staphylococcus aureus and *Enterobacter cloacae*. 

After 3 years on hemodialysis, he presented with paroxysmal abdominal pain and progressive abdominal distension, evolving over 2 months. He reported no changes in bowel habits, nausea, or vomiting. There was no deterioration in general condition. 

On physical examination, the abdomen was distended with dullness on percussion. The umbilicus was flattened, with mild generalized tenderness but no guarding. There was no visceromegaly or collateral venous circulation. 

Laboratory tests revealed an elevated C-reactive protein level, while leukocyte count and blood cultures were normal. Liver enzymes were within normal limits, and hepatitis serology was negative. Tumor markers including ACE, CA19-9, CA125, and alpha-fetoprotein were negative. 

**Figure 1.**
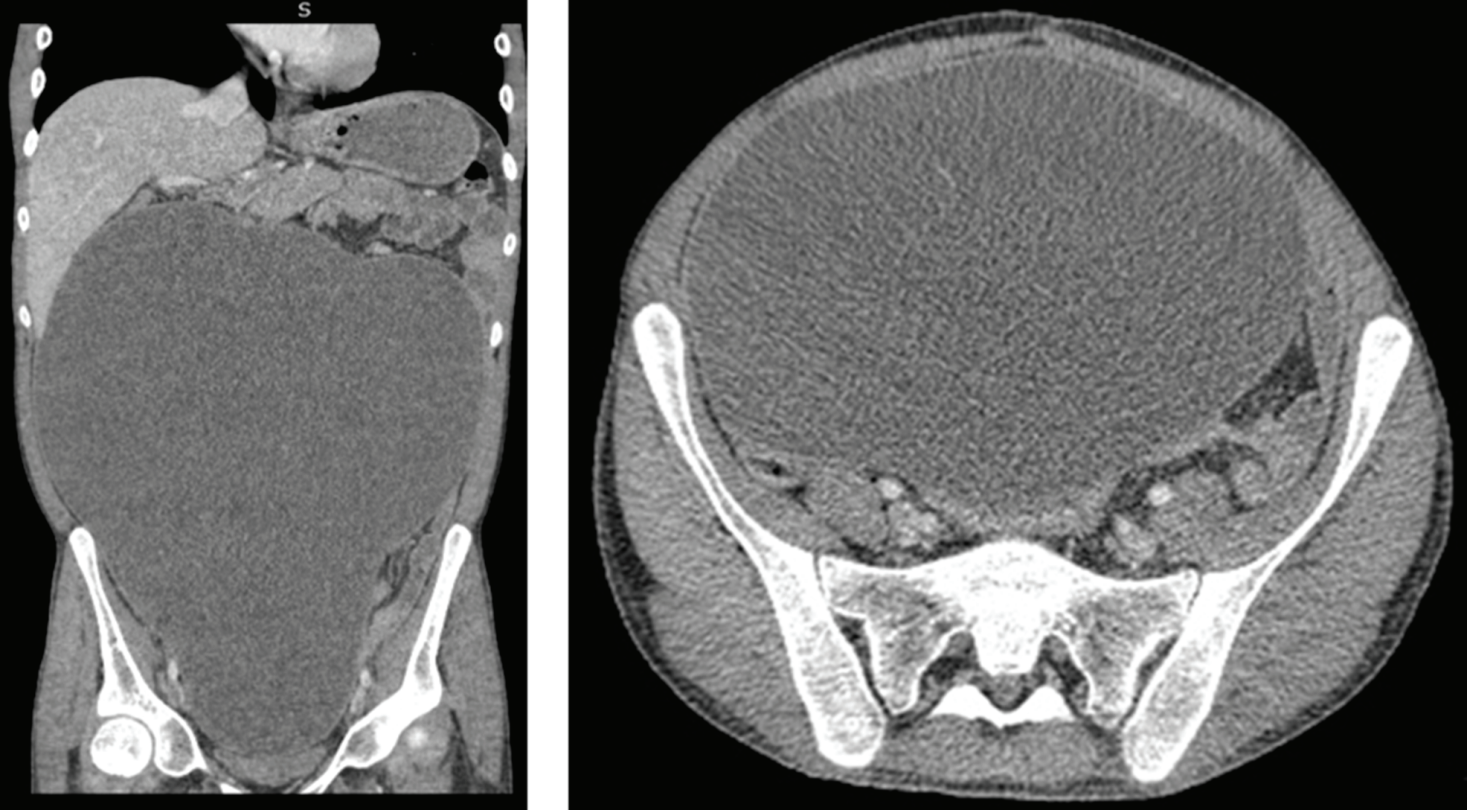
Abdominal computed tomography scan showing a giant encapsulated cystic mass occupying the entire peritoneal cavity and compressing adjacent organs.

Abdominal ultrasound revealed abundant encapsulated fluid extending from the epigastrium to the pelvis. Computed tomography (CT) confirmed a large encapsulated intra-abdominal collection compressing adjacent organs without other detectable lesions (Figure 1). 

Diagnostic paracentesis yielded an exudative serohematic fluid. Cytologic examination demonstrated poor cellularity without evidence of malignancy. 

**Figure 2.**
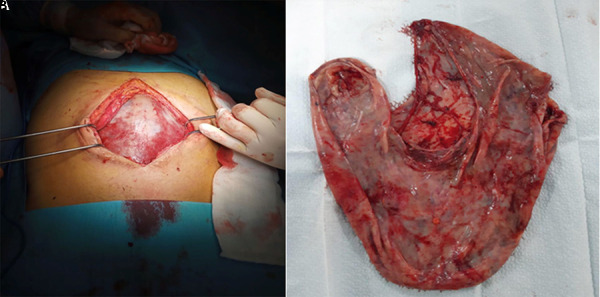
A: Intraoperative view showing a giant cystic lesion after midline laparotomy. B: Resected cystic specimen after complete surgical excision.

Surgical exploration via midline laparotomy revealed a giant cystic lesion (Figure 2A) adherent to the peritoneum of Douglas. No solid component was identified. No obvious vascular pedicle or clearly identifiable site of origin from the visceral peritoneum was observed. The lesion appeared to arise from the mesenteric peritoneal surface and was completely resected without macroscopic peritoneal breach (Figure 2B). Inspection of the remaining abdominal cavity revealed only minor adhesions, which were released. Approximately 4 L of serohematic fluid were drained. The postoperative course was uneventful, and the patient was discharged on day 10. 

**Figure 3.**
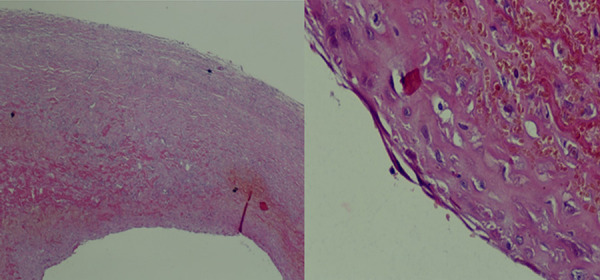
Histopathological examination showing a fibrous cyst wall lined by flattened mesothelial cells, consistent with a benign mesenteric cyst of mesothelial origin (hematoxylin and eosin staining).

Histologic examination revealed a fibrous-walled cyst lined by a single layer of cuboidal to flattened mesothelial cells, well-vascularized, with no evidence of malignancy, consistent with a benign cystic mesothelioma (Figure 3). 

## Discussion 

Mesenteric cysts are rare benign intra-abdominal lesions, with an estimated incidence of approximately 1 : 150,000 in adults and a female predominance [3, 4]. They most commonly arise from the ileal mesentery but may occur anywhere along the mesentery from the duodenum to the rectum [1, 3, 5]. Histopathologically, mesenteric cysts are classified into several subtypes, including cysts of lymphatic, mesothelial, enteric, urogenital, dermoid origin, and pseudocysts [6]. Their clinical presentation is highly variable, ranging from incidental findings to abdominal pain and progressive distension [7], and complications may occur in up to one-third of cases [3]. 

Preoperative diagnosis relies mainly on imaging modalities such as ultrasonography and CT [8]. In our patient, ultrasonography failed to accurately characterize the lesion and revealed only abundant intra-abdominal fluid, likely due to the exceptionally large size of the cyst. 

Although rare, intraperitoneal pseudocysts have been described in patients undergoing peritoneal dialysis, particularly following episodes of peritonitis [2, 9]. Less than 20 cases have been reported, with only 2 diagnosed after discontinuation of peritoneal dialysis [10, 11]. To our knowledge, mesenteric cysts of mesothelial origin occurring during hemodialysis have been reported only once previously, in a patient who developed a giant cystic mesothelioma 4 months after switching from peritoneal dialysis to hemodialysis [8]. In contrast, the delay in our case was considerably longer, with diagnosis occurring 3 years after the initiation of hemodialysis. 

Several mechanisms may contribute to the development of mesenteric cysts in this setting. Recurrent episodes of peritonitis appear to play a major role [12]. Peritoneal cystic mesothelioma has also been associated with prior abdominal surgery and chronic inflammatory conditions [13]. In our patient, persistent systemic inflammation related to long-term hemodialysis may have acted as an additional contributing factor. 

The main differential diagnosis in long-term peritoneal dialysis patients presenting with abdominal complications is encapsulating peritoneal sclerosis (EPS), a rare but severe complication typically occurring after 3 – 5 years of peritoneal dialysis. EPS is characterized by diffuse peritoneal thickening, fibrosis, calcifications, and bowel encapsulation. Notably, most cases are diagnosed after discontinuation of peritoneal dialysis and transition to hemodialysis or within 2 years following kidney transplantation [14]. Although our patient also developed the lesion after switching to hemodialysis, the radiological and histopathological findings were clearly distinct. In contrast to the diffuse fibrotic remodeling observed in EPS, our case demonstrated a well-circumscribed mesothelial-lined cyst without evidence of diffuse peritoneal thickening or bowel encapsulation. While chronic peritoneal inflammation and recurrent peritonitis may represent shared pathogenetic factors, these two conditions remain distinct pathological entities. 

Complete surgical excision remains the treatment of choice for mesenteric cysts and can be performed by either laparoscopic or open approaches [3]. Although conservative treatments such as image-guided drainage have been proposed, their efficacy and safety remain uncertain [15]. After resection of benign cystic mesothelioma, recurrence rates range from 30 to 50%, with reported intervals from a few months to several years [13]. Malignant transformation is rare, occurring in less than 3% of cases [16]. 

## Conclusion 

Mesenteric cyst formation is a rare complication of peritoneal dialysis. The originality of this case lies in the occurrence of a benign cystic mesothelioma 3 years after the switch to hemodialysis. Although the underlying mechanisms remain unclear, previous peritoneal dialysis, recurrent episodes of peritonitis, and chronic inflammation may represent predisposing factors. This case highlights the importance of long-term clinical surveillance in patients previously treated with peritoneal dialysis, even after transition to hemodialysis. While regular clinical follow-up with careful abdominal examination is recommended, imaging studies such as abdominal ultrasound or CT should be reserved for patients presenting with concerning signs, including unexplained abdominal distension, persistent inflammatory markers, or atypical abdominal symptoms, to enable timely diagnosis and prevent potential complications. 

## Informed consent statement 

Written informed consent was obtained from the patient for the publication of this case report and the accompanying images. 

## Authors’ contributions 

Sarra Hadded: Data collection, manuscript writing, patient follow up, final approval. Chayma Soltani: Assistance in data collection, literature review, manuscript revision, final approval. Wael Ferjaoui: Surgical management, intraoperative data, figure preparation, approval. Yosra Ben Ariba: Clinical follow-up, laboratory data, manuscript review, final approval. Jannet Laabidi: Supervision, critical manuscript revision, conceptual guidance, final approval. 

## Funding 

None. 

## Conflict of interest 

The authors declare no conflict of interest.
